# Second-Hand Smoke Exposure and Risk of Lung Cancer Among Nonsmokers in the United States: A Systematic Review and Meta-Analysis

**DOI:** 10.3390/ijerph22040595

**Published:** 2025-04-10

**Authors:** Safa Elkefi, Gabriel Zeinoun, Achraf Tounsi, Jean-Marie Bruzzese, Corina Lelutiu-Weinberger, Alicia K. Matthews

**Affiliations:** 1 School of Nursing, Columbia University, New York, NY 10027, USA; 2 Automatic Data Processing, New Jersey, NJ 07932, USA

**Keywords:** second-hand smoke, lung cancer, cancer risk, tobacco, nonsmokers

## Abstract

This study aims to explore the link between exposure to tobacco smoke among nonsmokers and the risk of lung cancer in the United States. We searched six databases for studies on second-hand smoke (SHS) and lung cancer following PRISMA guidelines. Following the random effects model and specific statistical methods, our meta-analysis analyzed studies based on SHS exposure type. A total of 19 eligible studies were included in the review and 15 in the meta-analysis. We covered exposure from parents (childhood), spouses and partners (household), and work-related exposure (colleagues), with higher risk among non-smoking children and domestic partners. Findings reveal a consistent link between SHS exposure and increased lung cancer risk for this population (exposure effect sizes: 1.05–3.11). Analysis of childhood SHS exposure reveals a distinct increased risk associated with parental exposure. For nonsmokers living with smoking spouses, there is a marked 41% increase in risk. Higher risk was associated with more and more prolonged SHS exposure. Exposure to SHS in the workplace shows a correlation with lung cancer risk. Our findings highlight increased SHS-related lung cancer risk, particularly among non-smoking children and domestic partners, intensifying with the amount and duration of exposure, indicating the significant impact of SHS within domestic environments.

## 1. Introduction

Second-hand smoke (SHS) is a significant public health threat comparable to the risks of active smoking [1]. One out of three people in the world are passive smokers [1]. In the United States (US), between 2015 and 2018, approximately 20.8% of nonsmokers have been exposed to SHS [2], as measured by the presence of cotinine in their blood [2]. Notably, 25.6% of individuals between 18 and 39 had been exposed to SHS, compared to 19.1% of individuals between ages 40 and 59 and 17.6% aged 60 and older [2]. SHS exposure was more prevalent among Black adults (39.7%) compared with Hispanic (17.2%) and White (18.4%) adults [2]. Conclusive scientific evidence documents that SHS is harmful to nonsmokers [3,4,5]. Numerous studies conducted across different continents supported the causality between SHS and lung cancer [6]. Furthermore, in adults, SHS has direct adverse cardiovascular issues, while long-term exposure can lead to the development of heart diseases, strokes, and sometimes death [5]. Exposure to SHS puts children at higher risk of sudden death syndrome, respiratory infections, worsening asthma, respiratory symptoms, and slowed lung growth [5].

Many research studies have investigated the link between SHS and being exposed to smoke at home and in the workplace. While smoke-free policies usually focus on public and work settings, fewer countries have implemented smoking exposure regulations in private or semi-private spaces, such as public housing and vehicles [7]. Considering the high likelihood of males smoking (fathers/spouses), children and women may benefit less from smoke-free measures in non-public spaces [7,8]. Many individuals encounter difficulties establishing rules prohibiting smoking within the household or reducing exposure to SHS emanating from adjacent residences [9] and often resort to measures (e.g., smoking only in some regions of the residence or smoking through open windows) that remain less effective [9]. Non-smoking households in multiunit housing face these particular challenges, as public smoking bans may not fully protect them from SHS [10]. Future research should also investigate the long-term effects of workplace (and other public place smoking bans, like bars and restaurants) smoking bans on lung cancer risk.

Prior studies have confirmed the causal link between SHS exposure and lung cancer [6,11,12]. Fewer have examined the contexts in which SHS exposure occurs and how these may impact risk differently. Understanding where individuals are most exposed—at home, work, or during childhood—is essential for designing targeted prevention strategies. Several meta-analyses and systematic reviews have evaluated the general association between SHS and lung cancer [6,11,12]. Nevertheless, the aforementioned studies lacked differentiation between exposure settings, thereby complicating the identification of specific locations that significantly contribute to lung cancer risk. This review enhances prior research through the following methodologies: (1) stratifying second-hand smoke (SHS) exposure by specific settings (such as childhood, household, and workplace) to ascertain which environments present the highest risk; (2) conducting a meta-analysis that isolates various types of exposure (such as childhood, household, and workplace) permitting a more accurate evaluation of risk variation across different contexts; and (3) addressing the heterogeneity prevalent in prior findings by accounting for varying exposure durations and intensities. By addressing this research gap, the current study offers pragmatic insights for policymakers and healthcare practitioners in prioritizing their public health initiatives aimed at mitigating SHS exposure.

Furthermore, this study is confined to the United States for several reasons. One is that smoking behaviors and patterns of SHS exposure exhibit considerable variation on a global scale, complicating the validity and feasibility of international comparisons. Additionally, the smoking regulatory frameworks, including smoke-free public spaces (e.g., workplace, public transportation) legislation and housing regulations, diverge substantially across nations. Focusing exclusively on US data guarantees a locally relevant policy context, which is not universally generalizable. Ultimately, public health recommendations necessitate a country-specific approach, as findings derived from a U.S.-centric perspective enable the formulation of highly contextualized policies and intervention strategies devoid of the complexities introduced by global regulatory disparities.

## 2. Materials and Methods

A literature review is conducted, followed by a meta-analysis based on a selection of these articles. Our systematic review of the literature followed the PRISMA guidelines (Preferred Reporting Items for Systematic Reviews and Meta-Analysis) [13,14].

### 2.1. Search Strategy

**Figure A1:**
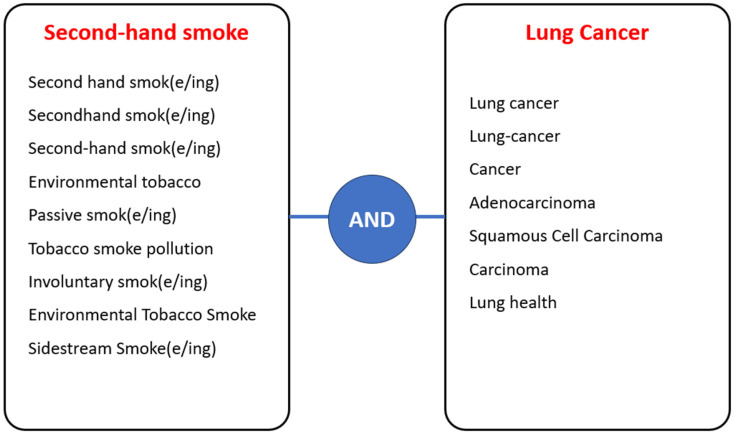
Keywords used in the search. An example of a mesh expression would be composed as follows; (second-hand hand smok OR sceond hand smoke OR second hand smoking) AND (Lung cancer).

We searched through six databases to gather pertinent publications published up to December 2023: Google Scholar, PsycINFO, Scopus, PubMed, Web of Science, and IEEE Xplore. These databases were selected based on their coverage of key research areas related to SHS exposure, lung cancer risk, and environmental health impacts [15]. For instance, PubMed provides biomedical and epidemiological research, ensuring coverage of clinical and public health studies on SHS and lung cancer. Scopus and Web of Science index high-impact journals across public health, epidemiology, occupational health, and environmental sciences, ensuring comprehensive coverage of peer-reviewed literature. PsycINFO captures research on behavioral aspects of SHS exposure, including risk perception and psychosocial impacts. IEEE Xplore includes air quality monitoring, environmental health, and occupational SHS exposure studies. Finally, Google Scholar captures studies that may not be indexed in traditional databases, ensuring search comprehensiveness. By selecting these databases, we ensured broad disciplinary coverage and maximized the inclusion of relevant studies. The search incorporated keywords relevant to SHS and lung cancer (Appendix A, Figure A1). Experts and librarians reviewed the keywords.

### 2.2. Inclusion Criteria

We included studies that (1) were empirical (quantitative or qualitative), (2) reported outcomes specifically related to lung cancer, (3) examined the association between SHS exposure and lung cancer risk, (4) were peer-reviewed, (5) were written in English, and (6) were conducted in the US. Studies focusing solely on other malignancies caused by tobacco exposure, such as head and neck cancers, were excluded given the focus of the present inquiry.

### 2.3. Study Selection

Among the selected studies, only those that reported quantitative findings on the association between SHS exposure and lung cancer risk were included in the quantitative analysis (meta-analysis). The selection was conducted in three stages, following PRISMA guidelines. First, following removal of duplicates, three independent reviewers screened titles and abstracts for relevance. Next, the remaining articles underwent a full-text review by the same three reviewers to assess eligibility. Finally, studies that met all inclusion criteria were selected for the systematic review and, where applicable, for the meta-analysis. Discrepancies at each step were discussed until a consensus was reached. If disagreements persisted, a fourth senior reviewer was consulted to make the final decision. This approach ensured that selection decisions were robust, unbiased, and consistent.

### 2.4. Data Extraction and Quality Assessment

The team collaboratively developed a data extraction form to capture the data for our research questions, which was pilot tested on five articles and refined iteratively (see Appendix B, Table A1).

Following the meta-analysis practices [16], we separated the study analysis based on the area of exposure reported (household, childhood, work, all types together) to prevent multiple weighting of the same study. We used the Joanna Briggs Institute’s (JBI) critical appraisal checklists to evaluate the risk of bias in each study [17].

### 2.5. Data Analysis

Following Masoumi et al.’s study [18], we conducted a meta-analysis to estimate the pooled effect size of second-hand smoke (SHS) exposure on lung cancer risk, using log-transformed odds ratios (ORs) with 95% confidence intervals (CIs). Given the expected heterogeneity among studies (differences in populations, exposure assessment methods, and study designs), we used a random-effects model, which assumes that individual studies estimate different but related actual effects rather than a single common effect [17]. This model was chosen over a fixed-effects model because it accounts for both within-study and between-study variability, making it more suitable when study characteristics vary significantly. The random-effects model ensures that our findings are more generalizable across different populations and study conditions, allowing for variability in the underlying effect size.

We applied the Hartung–Knapp–Sidik–Jonkman (HKSJ) adjustment to improve confidence interval accuracy, which is particularly useful in meta-analyses with moderate-to-high heterogeneity and a small-to-moderate number of studies. Compared to conventional random-effects models, HKSJ (1) adjusts for uncertainty in variance estimation, reducing the risk of overly narrow confidence intervals, which can lead to overconfident conclusions; (2) reduces type I error rates, making it more reliable in scenarios where standard random-effects models might overestimate precision; (3) and provides more accurate estimates when the number of included studies is relatively small, often in exposure-based epidemiological meta-analyses.

We assessed statistical heterogeneity using Cochran’s Q and I^2^ statistics, with I^2^ values classified as low (≤25%), moderate (26–50%), and high (>50%) heterogeneity [18,19]. High heterogeneity suggests substantial variability in effect sizes across studies, which justifies using a random-effects model. Publication bias was evaluated using funnel plots and Egger’s test [19,20,21,22] to determine whether small-study effects influenced our findings. To assess the robustness of our results, we performed sensitivity analyses using the leave-one-out method, systematically excluding each study one at a time to evaluate its influence on the overall effect estimate. Additionally, we explored potential sources of heterogeneity through subgroup analyses based on exposure type (childhood, household, workplace) and study design. More details are in Appendix A.

## 3. Results

### 3.1. Study Identification

**Figure 1:**
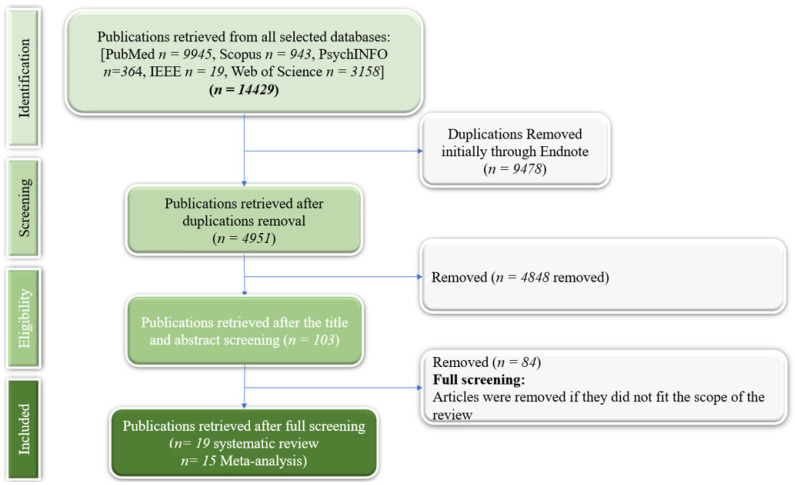
PRISMA flowchart.

We first identified 54,429 articles (Figure 1). After screening, 19 studies were eligible for our review (Table A2, Appendix C), and 15 were eligible for the meta-analysis (Appendix E).

### 3.2. Study and Participant Characteristics for the Systematic Review

Appendix B (Table A1) summarizes the included studies’ baseline characteristics, published between 1983 and 2023. Study designs varied: nine were case–control, five were cohort, two were cross-sectional surveys, two were longitudinal surveys, and one was qualitative.

Four studies had national samples, while others were conducted in different US regions: Northeast (*n* = 5); Midwest (*n* = 5); South (*n* = 6); and West (*n* = 4). Most studies (18/19) adjusted for gender, and seven included only women.

Sample sizes varied, with case–control studies ranging from 134 to 1338 participants, cohort studies from 810 to 133,385, cross-sectional surveys from 47 to 49,569 participants, and longitudinal studies from 19,286 to 41,632 nonsmokers. An interview-based study had 537 participants.

Participant demographics also varied. Six studies did not adjust for race, two did not account for age, and three focused only on women, adjusting for age. Three studies reported findings for specific populations (White women, Black women), all adjusting for age. Six studies did not report participants’ socioeconomic status.

### 3.3. Quality Assessment for the Systematic Review

Following the quality assessment results, we deemed all studies sufficiently adequate in quality to be included in the systematic review (Appendix D). All studies surpassed the quality threshold (i.e., eight in JBI).

### 3.4. Systematic Review Findings on the Risk of Lung Cancer per Exposure Area to SHS

#### 3.4.1. Lung Cancer Risk Associated with Overall Exposure to SHS (All Sources Together)

Eight studies reported findings related to overall SHS exposure (see Appendix Cand Appendix E). Three studies found a positive association with lung cancer risk [23,24,25], with effect sizes ranging from 1.05 to 3.11. Only one study found a non-significant relationship [26]. Only one of these studies focused on Black populations’ exposure to SHS and was limited to Black women. One study showed that the risk of exposure is higher starting from five years of continuous exposure (OR = 1.28) [24], while another study found that lifetime exposure to SHS was associated with increased lung cancer risk (OR = 1.14) [25]. Another study investigating long-term exposure showed that the more non-smoking people are exposed to tobacco smoke, the higher the risk of lung cancer (Exposure <20 years: OR = 1.09; Exposure 20–38 years: OR = 1.21; Exposure >38 years: OR = 1.32) [27]. For multiple types of SHS exposure, a combined exposure of a minimum of 10 years during childhood, 30 years during adulthood, and 20 years of work exposure resulted in the highest risk in (OR = 1.76) [28]. It is noteworthy that the study by Bastian et al. showed that women veterans had higher rates of exposure to passive smoke but did not show a higher adjusted risk for lung cancer compared to non-veterans [29].

#### 3.4.2. Lung Cancer Risk Associated with Childhood Exposure to SHS

Nine studies reported findings related to childhood exposure to SHS. We included six of these in the meta-analysis.

##### Both Parents Smoking

Seven of the studies reported findings related to both parents smoking (see Appendix C and Appendix E), but only four were subsequently included in the meta-analysis. Two studies investigated both parents’ history of smoking, both of which showed a positive correlation between SHS exposure and lung cancer risk [25,30]. Additionally, a study by Janerich et al. found that people who had prolonged exposure of up to 21 years of age had twice the risk of lung cancer [25]. The findings by Tyc et al. showed that 58% of the parents whose children had lung cancer smoked inside the home, with higher exposure among older children [31]. Another study revealed a higher risk of lung cancer with a higher intensity of exposure, although the associations were not significant [26]. Similarly, no clear associations were found in the study by Bastian et al. [29].

##### Mothers or Fathers Smoking

Three studies assessed the difference in children’s SHS exposure from mothers and fathers [23,30,32]. They each showed that children whose mothers smoked were at higher risk of lung cancer (OR = 1.60–2.92) compared to the risk associated with fathers smoking (OR = 1.04–2.89) [23,30,32].

#### 3.4.3. Lung Cancer Risk Associated with Household Exposure to SHS

Fourteen studies examined the risks related to household exposure, particularly by spouses and partners. We included 11 in the meta-analysis, which indicated that significant lung cancer-related risks were associated with passive smoking among married couples [24,33] and showed that women who were married to husbands who smoked more than forty cigarettes per day or who were exposed to the smoke of at least 20 cigarettes per day at home showed a doubled risk compared to non-exposed women. A study comparing household exposure to childhood SHS exposure showed that high levels of environmental tobacco smoke exposure in adulthood among non-smoking women increased their lung cancer risk by approximately 30% [26]. In contrast, the same study noted no consistent elevated risk among the same population for childhood or workplace exposure [26]. Rates of lung cancer deaths were 20% higher among spouses with smoking husbands compared to never-smokers, interestingly with a higher relative risk among women whose husbands smoked compared to when the wives were smoking (RR 1.6 vs. 1.1) [34].

#### 3.4.4. Lung Cancer Risk Associated with Workplace Exposure to SHS

Seven studies covered work-related SHS exposure, of which four were included in the meta-analysis. All the included studies showed a positive association with lung cancer risk, with a higher risk associated with more prolonged exposure [27,28,35]. For instance, a study by Ref. [27] reported that the risk of lung cancer increased from OR = 1.04 to OR = 1.20 and OR = 1.26 for less than eight years, 8 to 20 years, and above 20 years of exposure, respectively [27]. Ref. [35] demonstrated significant health risks from exposure to SHS in restaurants and bars (OR = 1.22). Rates of lung cancer death for servers and patrons were well above acceptable levels due to SHS exposure in these settings [35]. Another study investigating occupational exposure among Black women showed that SHS significantly increased lung cancer risk (sHR = 1.93, *p* = 0.006) [36].

### 3.5. Meta-Analysis Results

Below, we report findings from the meta-analysis conducted per exposure area (household, childhood, and workplace-related exposures) for the 15 included studies. Four studies were excluded for different reasons; one reported findings related to the occurrence of secondary lung cancer, which can represent a bias to our findings on the risk of primary lung cancer [37]. Studies focusing on secondary (metastatic) lung cancer were excluded because their outcomes do not represent the risk of developing lung cancer due to SHS exposure but rather the spread of cancer from other primary sites. Other studies reported their outcomes by comparing the risk among passive smokers compared to active smokers instead of non-smoking people who are exposed vs. not exposed to SHS [29,31,33]. While these studies provide insights into lung cancer risk, the way the data are presented does not allow use for meta-analysis purposes [29,31,33].

#### 3.5.1. Lung Cancer Risk Associated with Overall Exposure to SHS

The combined effect size in this analysis is 1.08. The proximity of the confidence interval to 1 indicates that, while there may be an effect, it might be of a lower magnitude than what could be considered impactful or definitive. Moreover, there is considerable heterogeneity among the studies, as indicated by an I^2^ of 67.08%, suggesting that while there is a general trend towards a positive effect, the studies vary significantly in the magnitude of that effect. Cochran’s Q statistic of 21.26 with 7 degrees of freedom reinforces this observation of notable heterogeneity among the studies, possibly due to various factors such as different study designs.

#### 3.5.2. Lung Cancer Risk Associated with Childhood Exposure to SHS

The combined effect size of exposure by both parents was OR = 1.40, suggesting a statistically significant association between the exposure or condition studied and the lung cancer risk. Despite this significant combined effect (*p* < 0.001), there is substantial heterogeneity among the studies (I^2^ = 84.77%), suggesting that while the studies are pointing in the same general direction (i.e., an effect exists), they differ significantly in the magnitude of that effect. Cochran’s Q statistic of 19.69 suggests low heterogeneity among the studies, which suggests that the studies are somewhat consistent. The sensitivity analysis suggests that all scenarios show high heterogeneity, indicating that the studies are quite different, regardless of which one is excluded. Although results change when excluding the different studies, the overall combined effect size always indicates a link between the nonsmokers’ risk of lung cancer and SHS exposure.

For mothers’ exposure, while there appears to be a considerable positive association between exposure and risk when the studies are combined, this effect is not statistically significant across the three studies included in this meta-analysis (OR = 1.40; *p* = 0.24). The studies have moderate heterogeneity (I^2^ = 28.48%), indicating that the study results are not drastically different. The sensitivity analysis suggested that excluding different studies changes the combined effect size and levels of heterogeneity significantly. The study by Olivio-Martson et al. [30] seems fundamentally different from the other two, as excluding it results in no observed heterogeneity among the remaining studies and lowers the combined effect size. That study is the only one suggesting a significant positive association between the risk of lung cancer and SHS exposure to fathers’ smoking.

For exposure to fathers’ smoking, the combined effect size from the three studies is 1.22 and the pooled effect is significant (*p* = 0.028). However, the studies have high heterogeneity (I^2^ = 72.04%), meaning the study results vary substantially. The Cochran’s Q of 7.15 with 2 degrees of freedom also suggests variability among the study outcomes. The sensitivity analysis shows that all combined effect sizes are more significant than 1, which generally supports the hypothesis that exposure to fathers’ smoking is linked to a higher risk of lung cancer among nonsmokers. Given the high effect sizes and significant findings, there seems to be a robust association between exposure to fathers’ smoking and increased lung cancer risk across different contexts. However, the significant heterogeneity when excluding different studies suggests that the exact magnitude of this risk may vary based on specific conditions or populations.

#### 3.5.3. Lung Cancer Risk Associated with Household Exposure to SHS

**Figure 2:**
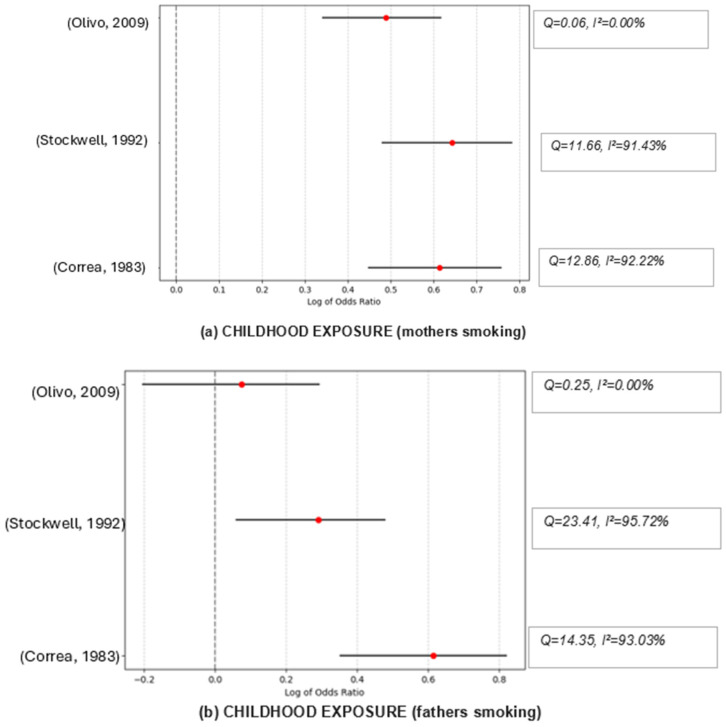

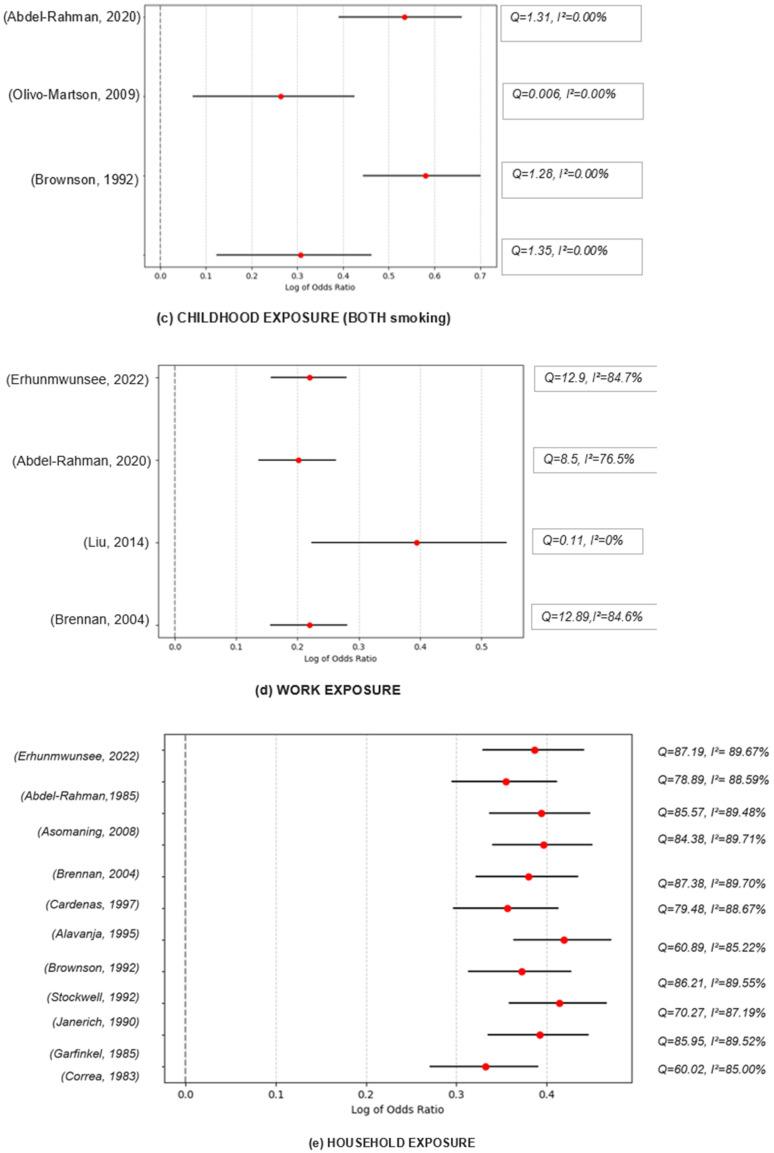
Funnel graphs for the excluded studies one at a time (sensitivity analysis). Childhood exposure (mothers smoking): Olivo, 2009 → [30], Stockwell, 1992 → [32], Correa, 1983 → [23]; Childhood exposure (fathers smoking): Olivo, 2009 → [30], Stockwell, 1992 → [32], Correa, 1983 → [23]; Childhood exposure (both smoking): Abdel-Rahman, 2020 → [38], Olivo-Marston, 2009 → [30], Brownson, 1992 → [26], Janerich, 1990 → [25]; Work exposure: Erhunmwunsee, 2022 → [36], Abdel-Rahman, 2020 → [38], Liu, 2014 → [35], Brennan, 2004 → [27]. Household exposure: Erhunmwunsee, 2022 → [36], Abdel-Rahman, 2020 → [38], Asomaning, 2008 → [39], Brennan, 2004 → [27], Cardenas, 1997 → [34], Alavanja, 1995 → [40], Wang, 1995 → [28], Stockwell, 1992 → [32], Janerich, 1990 → [25], Garfinkel, 1985 → [24], Correa, 1983 → [23].

When looking at the combined effect of all the studies that reported household SHS exposure, we found that overall household exposure is associated with 41% more risk when compared to unexposed individuals (*p* < 0.001). The high Q value of 51.57 indicates substantial heterogeneity among the results of the different studies. Despite the significant association, the high heterogeneity (I^2^ = 80.61%) suggests that the study outcomes differ substantially. This variation could be due to differences in study populations, methods, exposures, or other factors. In sensitivity analyses, where each study was excluded to examine its influence on the overall effect estimate, we observed consistent results that support the association between SHS exposure and an increased risk of lung cancer. The combined estimates ranged from 1.39 to 1.52 when each study was excluded, indicating that no single study disproportionately influenced the overall effect estimate. For instance, when excluding the study by Correa et al. [23], the combined effect size was OR = 1.39, indicating a significant association between SHS exposure and lung cancer. Similarly, excluding the study by Ref. [24] yielded a combined effect size of 1.48. This pattern was consistent across all excluded studies, underscoring the robustness of our findings. The I^2^ values ranged from 69.58% to 82.87%, suggesting substantial heterogeneity. It is important to note that Cochran’s Q statistic was significant in all cases, confirming the presence of heterogeneity (see Figure 2).

#### 3.5.4. Lung Cancer Risk Associated with Work Exposure to SHS

The combined effect size for the association between SHS exposure in workplaces and lung cancer risk among nonsmokers was OR = 1.24, indicating an increased risk of lung cancer associated with SHS exposure. Moderate heterogeneity among the studies (I^2^ = 41.05%) does not undermine the overall conclusion (since the *p*-value for Cochran’s Q suggests this variability could be due to chance). The moderate heterogeneity also indicates that the strength of this effect might vary slightly among different studies or populations. An I^2^ of 41.05% represents moderate heterogeneity.

## 4. Discussion

In this study, we investigated the impact of SHS exposure on lung cancer risk among nonsmokers. Our findings suggest a demonstrated association, with more prolonged exposure (>5 years) resulting in higher risk. These results align with the literature on the risks of SHS [6,41].

### 4.1. Childhood Exposure to SHS as a Risk of Lung Cancer

Our study showed that a higher risk of lung cancer was associated with both parents smoking [25,30]. This finding is consistent with previous studies on how parental smoking could harm children’s respiratory health [42,43]. Even when only one of the parents was smoking, the risk persisted [23,30,32]. However, mothers’ smoking resulted in a higher risk of lung cancer [23,30,32]. The time children spend with their mothers tends to be longer than that spent with fathers, which may explain this result [44,45]. Research has shown that time spent with a smoking parent significantly correlated with worse health outcomes in children [46].

Despite these findings, our sensitivity analyses revealed high heterogeneity among studies examining childhood SHS exposure. This heterogeneity persisted even after excluding individual studies, suggesting that the observed differences cannot be attributed to a single outlier. Several factors may explain this variability, such as differences in exposure measurement. For instance, some studies quantified SHS exposure using parental smoking status alone, while others considered intensity (cigarettes/day or pack-years) and duration (years of exposure). The observed heterogeneity across studies could also be related to the variability in study designs. For instance, case–control and cohort studies may yield different risk estimates due to differences in how participants are selected and recall bias in retrospective assessments. Also, it could be related to the recall bias in childhood exposure reporting. For instance, since lung cancer has a long latency period, exposure recall may be less precise, particularly in studies relying on self-reported childhood exposure from adults.

### 4.2. Household Exposure to SHS as a Risk of Lung Cancer

Our study revealed that women who do not smoke but are considerably exposed to tobacco smoke from their spouses (i.e., several cigarette packs/year and time) are at more risk of developing lung cancer compared to women not exposed at all [24], which is consistent with previous epidemiological studies [47]. Previous research has suggested that men have a higher risk of developing lung cancer [48]. Although previous studies do not support the higher female susceptibility to tobacco-related lung cancer, it is noteworthy that a study by Cardenas et al. found that exposure to spouse risk results in a higher chance of lung cancer among women compared to men [34]. This result suggests that while women might not be inherently more susceptible to tobacco-related lung cancer from personal smoking, they could be more vulnerable to the effects of SHS from their spouses compared to men. Further investigation is needed to validate this result.

Additionally, some studies showed that household exposure was associated with worse lung cancer outcomes than childhood and workplace exposures [24,26]. The authors elucidated two potential explanations. Firstly, the passive smoke-related risk of lung cancer during childhood may decline by adulthood, especially in the absence of adulthood exposure [26]. Secondly, there is potentially low reliability in the quantitative measures (both intensity and duration) of passive smoke exposure during childhood, rendering the assessment of lung cancer risk attributable to childhood passive smoke exposure incredibly challenging [26,49,50,51]. The same issue was attributable to the measures used to self-report exposure in the case of workplace exposure [52].

### 4.3. Workplace Exposure to SHS as a Risk of Lung Cancer

Our findings indicated a positive association between workplace-related SHS exposure and lung cancer risk, which strengthens with prolonged exposure [27,28,35]. It is noteworthy that although the association is positive, only the study by [35] investigated exposure to a specific environment of work (restaurants and bars), and one study by [36] reported associations for a specific population (Black women). All other studies reported general occupational exposure findings among all participants [27,28,36]. Additionally, no studies compared the exposure risk across different types of jobs. Research has differentiated the SHS exposure levels based on job type: indoor vs. outdoor, number of employees in the workplace, number of smoking employees, and field of occupation (mining, tourism, manufacturing, etc.) [53]. Considering that the difference in jobs may result in different risk outcomes, future research should incorporate this classification into workplace exposure assessment.

### 4.4. Practical Implications

Our results emphasize the importance of implementing efforts that minimize nonsmokers’ exposure to SHS at home and in the workplace. Regarding reducing risk for home SHS, clinicians could incorporate standardized questions about SHS exposure, particularly household exposure, into routine patient history intake, especially for individuals with no personal history of smoking, including children and adolescents. Relatedly, it is essential to consider expanding lung cancer screening eligibility criteria to include non-smoking individuals with significant SHS exposure, particularly those with additional risk factors. Prevention of SHS and potential subsequent lung cancer remains a critical public health priority and, based on findings from this study, family smoking cessation interventions may also be appropriate to raise awareness among household members and promote harm reduction strategies.

Public health efforts should focus on educating non-smoking women about the negative impacts of household SHS exposure, emphasizing the importance of establishing smoke-free zones within the home. One of our studies indicated that SHS exposure during childhood, particularly from both parents, increases lung cancer risk. However, the risk from childhood exposure may decline by adulthood if exposure does not continue, underscoring the need to protect children from SHS to reduce long-term cancer risk.

Workplace SHS exposure also presents a significant risk, particularly in service industries such as restaurants, bars, and casinos, where smoking remains relatively more prevalent. Policies are needed and/or enforced through strict accountability specifications for employers to implement stricter smoke-free workplace policies, create designated outdoor smoking areas, and ensure adequate ventilation when exposure is difficult to prevent. Additionally, further research is needed to assess the variation in SHS exposure across different job types, neighborhoods, and workplace environments, as factors such as the number of smoking colleagues and shared workspace design that may influence risk levels.

Societal responsibility in enforcing smoke-free policies should be prioritized at a broader level. Multiunit housing regulations should be strengthened and enforced to prevent SHS infiltration between residences through shared ventilation systems and common spaces. Expanding smoke-free policies to include outdoor public spaces like parks, bus stops, beaches, and restaurant patios could further protect nonsmokers. Public awareness campaigns should be implemented at the community level to educate individuals about the dangers of SHS exposure and the importance of complying with smoking restrictions. By integrating individual responsibility with policy enforcement, a comprehensive approach to SHS reduction can be achieved, ultimately reducing lung cancer risk in nonsmokers.

### 4.5. Limitations

The are some limitations to this review that we should acknowledge. Considering the differences in laws related to smoking in different countries, we excluded studies focusing on countries other than the US, which limits the generalizability of our findings. Additionally, we excluded articles written in languages other than English, which may have resulted in missing some studies. Furthermore, several included studies examined socioeconomic and racial/ethnic disparities in SHS exposure and lung cancer risk. However, not all studies accounted for SES and racial factors, similarly limiting our ability to conduct a detailed pooled comparative analysis across these demographic variables. Some studies adjusted for income, education, and housing status, while others only stratified by broad racial groups without accounting for socioeconomic factors. A few studies examined occupational class and workplace exposure disparities, but methods varied significantly, making direct comparisons difficult. Several studies did not adjust for SES at all, meaning potential confounding effects remain unaccounted for in some findings. While some studies used the number of packs/years to evaluate the high exposure, others considered the number of years exposed to smokers. Effectively measuring SHS is crucial to offering precise, timely, and cost-efficient ways of evaluating exposure intensity. In addition, many included studies relied on self-reported SHS exposure, subject to recall bias and misclassification errors. Individuals may underestimate or overestimate their exposure based on memory, leading to potential measurement inaccuracies. Studies using biomarker-based SHS assessment (e.g., cotinine levels) generally reported stronger associations between SHS and lung cancer risk, suggesting that self-reported data may dilute actual risk estimates. Also, the studies included in this review may be biased toward publishing significant findings, while studies with null or weak associations may be underreported. Certain studies had non-representative samples (e.g., hospital-based case–control studies), which may not reflect broader population-level effects. Finally, while the overall meta-analysis showed a positive association between SHS exposure and lung cancer risk, some studies reported weaker or non-significant associations. Possible explanations may include exposure misclassification, not adequately adjusting for active smoking, and variability in the exposure assessment methods.

## 5. Conclusions

This study reinforces the strong association between SHS exposure and lung cancer risk, emphasizing the need for comprehensive interventions to limit exposure in households, workplaces, and public spaces. The findings support stricter SHS policies, mainly through smoke-free housing regulations, workplace protections, and the expansion of outdoor smoke-free areas.

Despite strong evidence linking SHS to lung cancer, several critical gaps remain that warrant further investigation. Future studies should explore longitudinal trends in SHS exposure to determine how cumulative and long-term exposure influences lung cancer risk. Prospective cohort studies integrating biomarker-based assessments (e.g., cotinine levels) would improve exposure measurement accuracy and strengthen causal inferences. Additionally, emerging research suggests that genetic predisposition may influence individual susceptibility to SHS-related lung cancer. Investigating gene–environment interactions could help identify high-risk populations and inform personalized prevention strategies.

Moreover, more research is needed to examine racial, ethnic, and socioeconomic disparities in SHS exposure and lung cancer risk. While some studies adjust for these factors, many do not consistently account for these important social determinants of health as predictors for SHS exposure and lung cancer, limiting the ability to conduct in-depth analyses. Future studies should incorporate biomarker-based SHS assessments to reduce reliance on self-reported exposure, which is prone to recall bias. Additionally, studies should compare SHS exposure risk across different workplace environments, focusing on factors such as industry type, ventilation conditions, and workforce smoking prevalence. By addressing these policies and research gaps, public health strategies can be further refined to prevent SHS-related lung cancer and enhance protective measures for at-risk populations.

## Appendix A. Search Query Mesh Words Used

**INCLUSION/EXCLUSION CRITERIA:** This study included publications that were (a) written in English, (b) used US data, (c) studied and quantified the link between SHS and risk of lung cancer among nonsmokers, and (d) used self-reporting of the degree and contexts of exposure. Quantitative and qualitative empirical studies were included. Any articles on exposure to smoke other than tobacco (frying, cooking, work smoke exposure) were excluded. We also excluded gray literature, editorials, and dissertations. Opinion papers and commentaries were not included either.

**DATA ANALYSIS DETAILS:** Our meta-analysis methodology was guided by a study conducted by Masoumi et al. [18]. We only included studies that reported primary lung cancer diagnosis outcomes (diagnosis or death) and the studies that reported point estimates (relative risk (RR), odds ratio (OR), or standardized incidence ratio (SIR)), or statistics that allowed us to generate these point estimates. Overall, we included 15 studies in the meta-analysis. The Results Section (Section 3) details why each of the four studies were excluded from our meta-analysis. Only point estimates indicating high SHS exposure based on the study’s findings were included in the analysis. We reported the point estimates and their 95% confidence intervals (CI), with significance levels (*p* < 0.05)

When calculating the combined effect size, we used the random effects model, the Hartung–Knapp–Sadik–Jonkman method, and the generic inverse-variance method. We calculated the combined effect size using a weighted average of the log-transformed odds ratios. After standardizing the effect size into OR/RR/SIR, we assessed the heterogeneity using I^2^ and Cochran’s Q statistics to assess the variability among the study findings. The *p*-value from Cochran’s Q test was provided as evidence of the homogeneity of the effect sizes. All analyses were conducted per subgroup based on the type of exposure. For I^2^, values below or equal to 25%, between 26% and 50%, and 51% or above suggest low, moderate, and high levels of heterogeneity, respectively [19].

We also conducted influence analyses using the leave-one-out method for sensitivity analysis. This helped us identify any outlier studies that could significantly impact our overall results and distort our pooled effect [20]. Funnel plots were generated to assess publication bias [21,22].

## Appendix B. Data Extraction Form

**Table A1:** Data Extraction Form.

	Variable	Definition
Study details	Title	Title of the study/research paper.
	Year	The year the study was published.
	Objective	The primary goals or questions the study aimed to address.
	Study design	The methodology or approach used in the study (e.g., randomized controlled trial, cohort study, case–control study).
	Sample size	The number of non-smoking participants or observations included in the study.
Participants demographics	Age	The age range of the study nonsmoking participants.
	Gender	The gender distribution of the study participants (e.g., male, female, other).
	Race	The racial or ethnic composition of the study participants.
	Socioeconomic status	The social and economic characteristics of the study population are often measured by income, education, and occupation.
Exposure details	Exposure areas	Exposure to second-hand smoke (household: spouses and co-habitants, childhood: parents or siblings, workplace, overall).
	Duration of exposure	The length of time participants were exposed to SHS.
	Intensity of exposure	The degree or amount of exposure to the risk factor.
Health outcomes	Stage of lung cancer	The lung cancer stage, typically stages I–IV or death, indicates the severity and spread of the cancer.
	Lung cancer types	The specific type of lung cancer diagnosed.
Geographic information		City or cities, region or regions where the study took place.
Key findings		The main results or conclusions drawn from the study.

## Appendix C. Systematic Review Results

**Table A2:** Systematic Review Results.

Study Details		Participant’s Details						Exposure Details					Health Outcomes		City, State	**Key Findings**
Ref	Objectives	Study Design	Sample Size	Age Range	Gender	Race	Socioeconomic Status	Household	Childhood	Work	Duration of Exposure	Intensity of Exposure	Stage of Lung Cancer	Type of Lung Cancer		
[23]	Determine the smoking habits of the parents and spouses of individuals diagnosed with lung cancer (smokers versus nonsmokers	Case–control study	1338 lung cancer patients	Not reported	**Non-smoking participants** -Males: 241 -Females: 151	Not reported	**Married:** -Male: 188 -Female: 155	X	X		long-term	Not reported	Not reported	Not reported	Louisiana, New Orleans	Nonsmokers married to heavy smokers and individuals with smoking mothers had a higher risk of lung cancer. However, there was no apparent link between paternal and maternal smoking among nonsmokers and the risk of lung cancer.
[54]	Investigate the impacts of long-term passive smoking among nonsmoking women (working vs. nonworking) dead of lung cancer by interviewing their relatives	Retrospective study based on interviews and review of death notices	537 nonsmoking women	Median age 71.8	Female wives	Not reported	Middle-income group, with the average family income for 1970 listed as $93,809 (in Erie County, Pennsylvania)	X			long-term	Not reported	Death	Not reported	Pennsylvania	Exposure to second-hand smoke over a long period results in an increased risk of cancer-related deaths among nonsmokers who are exposed. Working women were more contaminated than women staying at home, an exposure hazard in the workplace. Results may be biased by external exposure to SHS that is not accounted for.
[24]	Examine the effects of second-hand smoke on the development of lung cancer. All individuals involved in the study were carefully confirmed through histologic examination of samples, and their non-smoking status and exposure to second-hand smoke were validated through interviews.	A case–control study was conducted in four hospitals.	134 nonsmoking women	40–59 years (33) 60–79 years (72) 80 or above (29)	women	Not reported	Middle class 75 Upper class 6 Lowe class 53	X			5-year and 25-year exposure	Based on the number of cigarettes	Microscopic proof of lung cancer	Adenocarcinoma was the most common type among cases, followed by large, squamous, and mixed or other cells.	New Jersey and Ohio	The research findings indicate a strong correlation between increased risk of lung cancer and prolonged exposure to spousal smoking at home. This correlation holds true even when adjusting for factors such as age, hospital, socioeconomic status, and year of diagnosis. Women married to spouses who smoke 40 or more cigarettes a day or who are exposed to the smoke of at least 20 cigarettes a day at home face a risk of lung cancer twice as high as those not exposed at all. Interestingly, the comparison of women based on the number of hours exposed to smoke in the last five years and in the last 25 years showed no significant increase in the risk of lung cancer.
[25]	**Determine the link between the risk of lung cancer and tobacco smoke exposure within the household**	**Case–control study and interviews**	**191 patients**	**Not reported**	**45 males** **146 females**	Not reported	Not reported	X	X		Childhood and adolescence, adulthood exposure, and lifetime exposure	Based on years and number of cigarettes	Diagnosis	Not reported	New York	Exposure to second-hand smoke (SHS) during childhood and adolescence for 25 years or more doubles the risk of lung cancer in the early decades of life (up to 21 years). However, exposure to SHS for fewer years showed no significant association with lung cancer. Additionally, exposure to a spouse’s smoking during adulthood did not appear to be linked to a diagnosis of lung cancer. Although data on exposure at work was collected, no significant results were observed.
[32]	Examine the correlation between exposure to environmental tobacco smoke and lung cancer risk among non-smoking women	Case–control study and interviews	210 patients	65% of adults under 65 years old and the rest above 65 years	Women	93% White among case patients;	51% were married/65% were above 12 grades education, and 18% were less than 8th grade	X	X		Childhood, adolescence, and adulthood years	Based on years and number of cigarettes	Diagnosis	Adenocarcinoma was the most common type among cases, followed by squamous cell and small-cell carcinoma.	Central Florida, Florida	It has been observed that the risk of lung cancer is higher among women who have never smoked in their lifetime but live in households with smokers. The elevated risks were consistently observed when exposure to household smoke occurred during adulthood. Women with non-adenocarcinoma lung cancers who reported high levels of exposure to household smoke had the most significant increase in risk. Additionally, there is suggestive evidence that prolonged exposure to tobacco smoke during childhood and adolescence may also be linked to an increased risk of lung cancer.
[26]	Analyze the connection between lung cancer and exposure to second-hand smoke during childhood and adulthood.	Case–control study and interviews	432 nonsmokers	30 to 84 years	women	White (due to small numbers of other racial/ethnic groups)	Not reported	X		X	Detailed for childhood (17 years and younger) and adulthood (18 years and older)	Based on years and number of cigarettes	Diagnosis	Adenocarcinoma was the predominant type; other types included squamous cell carcinoma, bronchioalveolar carcinoma, and small cell carcinoma.	Missouri	Exposure to high levels of environmental tobacco smoke in adulthood has been shown to increase the risk of lung cancer among nonsmokers by around 30%. However, there does not appear to be a consistently elevated risk associated with childhood exposure or exposure in the workplace.
[40]	Determine the population-attributable risks (PAR) for lung cancer in nonsmoking and long-term ex-smoking women, considering various risk factors such as environmental tobacco smoke, dietary intake, and other exposures.	Population-based case–control study	618 patients	30 to 84 years, with people between 75 and 84 years (48%)	women	White	47% married/39% less than 12th grade	X		X	Not reported	Not reported	Diagnosis	The major cell type was adenocarcinoma, followed by squamous cell, bronchoalveolar, small cell, and other types.	Missouri	SHS is the 2nd most predicted cause of lung cancer diagnosis in nonsmokers
[34]	Explore the impact of environmental tobacco smoke (ETS) on lung cancer mortality prospectively in the US.	Prospective cohort study	114,286 female and 19,549 male never-smokers married to smokers, compared with about 77,000 female and 77,000 male never-smokers whose spouses did not smoke.	30 years and above	Female and male	95.4% white	Education (11.1% have less 12th grade levels)	X			long-term	Number of cigarettes, years in marriage (exposure) and pack-years of exposure	Death	lung cancer of all types	nationwide in the United States, encompassing participants from all 50 states, the District of Columbia, and Puerto Rico.	Women whose husbands ever smoked had a 20% higher risk of lung cancer death compared to those married to never-smokers, with a relative risk (RR) of 1.2. For men who never smoked and had wives who smoked, the relative risk was 1.1 (CI = 0.6–1.8). These findings align with the EPA’s estimate that spousal smoking increases lung cancer risk by around 20% in never-smoking women.
[31]	Examine parental smoking behaviors and parent-reported exposure to Environmental Tobacco Smoke (ETS) among children treated for cancer.	Surveys	47 children and their parents	10 and 18 years.	57.4% male, 42.6% female.	78.7% Caucasian, 21.3% African American.	38.3% from upper-middle and 61.7% from lower-middle socioeconomic levels				Current and historical exposure as reported by parents	number of cigarettes smoked around the child	Diagnosis	Not reported	Memphis, Tennessee	According to this study, nearly 72% of parents admitted to smoking around their children, and almost 58% smoked inside their homes. The study also found that children who smoked or had a history of smoking had more exposure to environmental tobacco smoke (ETS) compared to non-smoking children. Additionally, older and Caucasian children were found to have higher levels of ETS exposure.
[33]	Describe patterns of ETS exposure and its association with lung cancer in women	Interviews and Cohort study	810 patients	31 to 91 years	Women	94% caucasian	Not reported	X	X	X	Mean years of exposure were 27 from a spouse, 19 from parents, and 15 from co-workers.	pack-years, divided into light, moderate, and heavy based on the number of cigarettes smoked by the source	Diagnosis	Adenocarcinoma, followed by squamous cell and carcinoid	Rochester, Minnesota	Over 95% of the women were exposed to tobacco smoke either personally or through ETS. There was significant exposure among nonsmokers, especially from spouses and parents. The study highlighted significant associations between ETS exposure and lung cancer among nonsmokers.
[27]	Examine the population-attributable risks (PAR) for various factors contributing to lung cancer among nonsmoking women and long-term ex-smokers	Population-based case–control study.	665 patients	20 to 79 years	women	Not reported	66.3% had higher education, and 16.5% had very low educational levels	X	X	X	long-term	Number of cigarettes and years of exposure	Diagnosis/death (next of kin)	Adenocarcinoma (76.1%), squamous cell (9.8%) and others	5 metropolitan areas: Atlanta, Georgia, New Orleans, Louisiana, Houston, Texas, Los Angeles, California, and San Francisco Bay Area, California.	Exposure to second-hand smoke and occupational hazards has been linked to an increased risk of being diagnosed with lung cancer. Research has shown that all three sources of exposure can lead to a higher risk of developing both adenocarcinoma and squamous/small cell carcinomas. When all three sources of exposure are considered together, a clear pattern of increased risk with a longer duration of exposure is evident for both types of lung cancer, with squamous and small cell carcinomas carrying a higher risk.
[39]	Study the correlation between SHS exposure and lung cancer, particularly focusing on the timing of the first exposure in relation to the participant’s age and adjusting for active smoking variables.	Case–control study	138 patients	mean 62 years old	56 makes and 82 females	129 whites and 9 other	Not reported	X	X	X	long-term	Number of exposure types (home, leisure, or work)	stage 1 and 2 (57) and stages 3 and 4 (75)	Adenocarcinoma, followed by squamous cells and others	Boston, Massachusetts	People who are exposed to second-hand smoke (SHS) before the age of 25 are at a greater risk of developing lung cancer compared to those who are first exposed to SHS after the age of 25. Research indicates that exposure to SHS during the period when the lungs are still developing up to the age of 25 has a significant impact.
[30]	Investigate the correlation between SHS exposure and lung cancer risk, particularly the impact of age at exposure, hypothesizing greater risk for exposures during lung growth period (0–25 years) compared to after age 25	A case–control study using self-reported SHS exposure	796 patients	mean 63 years old	369 males and 427 females	Not reported	151 had less than high school levels, and 57 had graduate degrees	X	X	X	Variable, categorized by age at first exposure (up to age 25, after age 25).	Number of exposure types (home, leisure, or work) and heaviness of the exposure (low, moderate, heavy)	Diagnosis	Not reported	Massachusetts	Individuals exposed to SHS, especially < 25, have a higher risk of lung cancer compared to those first exposed after age 25. The study supports the hypothesis that SHS exposure during lung growth periods leads to a greater risk of lung cancer in adulthood. Findings suggest a potential dose–response relationship, with higher risk associated with exposure in both work and leisure settings compared to one or none.
[28]	Investigate the connection between the occurrence of lung cancer and exposure to passive smoking during childhood, adulthood at home, and in the workplace.	Prospective cohort study	36135 patients	50 to 79 years	Not reported	83.37% Whites, 7% Black, 3.88% Hispanic, 4.23% Asian	4.49% had less than a high school degree, and 35.78% had some college	X		X	Mean years of exposure were 27 from a smoking spouse, 19 from parents, and 15 from co-workers.	Categorized based on the source smoker’s consumption (light: less than one-half pack per day; moderate: one-half to one pack per day; and heavy: more than one pack per day).	Stages III and IV for some proxy interviews to reduce survival bias.	Adenocarcinoma, squamous cells, unclassified non-small cell lung cancer (NSCLC), small cells, or carcinoids.	Rochester, Minnesota	In individuals who have never smoked but have been exposed to environmental tobacco smoke (ETS), the rates of exposure were found to be high from various sources, including 27 years from a spouse, 19 from parents, and 15 from co-workers. ETS exposure was found to be significant across all major subtypes of lung cancer in nonsmokers. There were statistically significant trends for adenocarcinoma, squamous, and small cell carcinoma among never-smokers with ETS exposure, showing a notable increase in adenocarcinoma and carcinoid tumors compared to those who have ever smoked. This study highlights the public health impact of tobacco smoke exposure and emphasizes the importance of eliminating smoking in both public and private spaces to reduce ETS exposure and the associated risks of lung cancer.
[29]	Examine the disparities in lung cancer rates in women veterans and non-veterans resulting from exposure to active and passive smoking.	longitudinal demographic, clinical, and laboratory data	983 veteran nonsmokers and 41632 non-veteran nonsmokers	50 to 79 years	women	veterans Whites 87% and veterans whites 84% veterans Hispanic 4% and non-veterans Hispanic 2.3% veterans black 9.1% and non-veterans black 7.1%	married non-veterans 62.2% and veterans 48.6%/college graduates veterans 46.8% and non-veterans 39.5%/income 75k or more veterans 14.2% and non-veterans 17.8%	X	X	X	long-term	Number of years and number of sources of exposure	Diagnosis	Not reported	All USA	Women who are Veterans had higher rates of passive smoking exposure; however, they did not demonstrate a higher adjusted risk for lung cancer compared to non-Veterans.
[38]	Identify the impact of SHS on lung cancer incidence and mortality among never smokers.	Cross-sectional baseline survey	49,569 participants.	55 to 74 years	Both sexes, the majority female (61.2%).	Mostly white (87.9%).	Not reported	X		X	long-term	Type of exposure	Diagnosis	Includes small-cell carcinoma, adenocarcinoma, bronchoalveolar carcinoma, squamous-cell carcinoma, and large-cell carcinoma	All USA	People who have never smoked but were exposed to second-hand smoke as adults are at a higher risk of being diagnosed with lung cancer later in life. Additionally, nonsmokers with a history of adult second-hand smoke exposure have a greater likelihood of dying from lung cancer.
[55]	Explore the cancer mortality risks associated with exposure to SHS.	Longitudinal, population-based, nationally representative health survey linked to mortality rates from the National Death Index database.	While specific for nonsmokers is not directly mentioned, the study evaluated a cohort of 25,794 US residents older than 19 years. Among them, 6508 (25%) had no exposure to tobacco smoke, which includes nonsmokers.	19 years and more (low exposure age mean 47.3, high exposure age mean 42.8)	The demographics included both males and females, but specific proportions were not provided in the text.	Race/ethnicity was categorized into five categories by NHANES investigators, but specific distribution was not detailed in the provided text.	Socioeconomic status was inferred through education and poverty-income ratio.	X		X	Not reported	Divided into two groups based on serum cotinine levels: low exposure (0.015 to 10 ng/mL) and high exposure (≥10 ng/mL).	Death	Not reported	All USA	Exposure to high levels of second-hand smoke (serum cotinine level ≥ 10) is significantly linked to higher mortality risks from several types of cancer, particularly lung cancer. However, low exposure to second-hand smoke has not shown a statistically significant association with cancer mortality.
[36]	To investigate the correlation between neighborhood disadvantage and lung cancer risk in Black never-smoking women.	Prospective cohort study using data from the Black Women’s Health Study.	37,650 never-smoker black women	20 to 70 years.	Females	Black/African American	Studied at both individual and neighborhood levels; specific socioeconomic data collected included individual income and educational attainment, alongside neighborhood socioeconomic status (SES) and neighborhood concentrated disadvantage.	X		X	Follow-up from 1995 to 2018.	Second-hand smoke exposure was quantified as being in a room with a smoker for at least one hour per day for 12 consecutive months; PM2.5 exposure and neighborhood-level exposures were based on residence data.	Diagnosis	Not reported	All USA	In this study, involving 37,650 individuals who had never smoked, 77 were found to have developed lung cancer during the follow-up period. The research revealed that for every ten-unit increase in the neighborhood concentrated disadvantage index, there was a 30% rise in the incidence of lung cancer (sHR = 1.30, 95% CI: 1.04, 1.63, *p* = 0.023). Exposure to second-hand smoke at work was found to elevate the risk of lung cancer significantly (sHR = 1.93, 95% CI: 1.21, 3.10, *p* = 0.006), whereas exposure at home and PM2.5 did not show significant associations with the disease.
[37]	Compare the total number of second primary lung cancer cases in lung cancer survivors who have never smoked with those who have ever smoked.	Population-based prospective cohort study using data from the Multiethnic Cohort Study (MEC) with follow-up through the Surveillance, Epidemiology, and End Results registry.	7161 participants had had initial lung cancer diagnosis, and 163 had secondary lung cancer	45 to 75 years	Among those who already had initial lung cancer, 4031 (56.3%) were male, and 3131 (43.7%) were female.	African American (16.3%) overall and 25.9% had initial lung cancer//white overall 23.1% and Initial lung cancer patients23.9% and 28.8% had secondary lung cancer.	Not reported	X		X	Not reported	Smoking status only	Diagnosis	initial primary lung cancer (IPLC) and second primary lung cancer (SPLC)	California and Hawaii	The cumulative 10-year incidence of second primary lung cancer (SPLC) after initial primary lung cancer (IPLC) was found to be equally high among survivors who never smoked and those who ever smoked. The standardized incidence ratio (SIR) for SPLC was significantly higher for never-smokers (14.50; 95% CI, 8.73–22.65) compared to ever-smokers (3.50; 95% CI, 2.95–4.12), indicating a notable risk of SPLC among never-smoking lung cancer survivors.

## Appendix D. JBI Results for Risk of Bias

**Table A3:** JBI Results for Risk of Bias.

	Similar Two Groups Recruited from the Same Population	Exposures Are Measured Similarly to Assign People to Exposed and Unexposed Groups	Exposure Measured in a Valid and Reliable Way	Confounding Factors Identified	Strategies to Deal with Confounding Factors Stated	Participants Free of the Outcome at the Start of the Study	Outcomes Measured in a Valid and Reliable Way	Follow-up Time Reported and Sufficient for Outcomes to Occur	Follow-up Was Complete, and if Not, Reasons Were Described and Explored	Utilized Strategies to Address Incomplete Follow-Up	The Analysis Used is Appropriate	Score
(Correa, 1983) [23]	1	1	1	1	1	1	1	0	0	1	1	9
(Miller, 1984) [54]	1	1	1	1	1	1	1	0	0	1	1	9
(Garfinkel,1985) [24]	1	1	1	1	1	1	1	1	1	1	1	11
(Janerich, 1990) [25]	1	1	1	1	1	1	1	0	1	1	1	10
(Stockwell, 1992) [32]	1	1	1	1	1	1	1	0	1	1	1	10
(Brownson, 1992) [26]	1	1	1	1	1	1	1	0	1	1	1	10
(Alanja, 1995) [40]	1	1	1	1	1	1	1	0	1	1	1	10
(Cardenas, 1997) [34]	1	1	1	1	1	1	1	0	1	1	1	10
(Tyc, 2004) [31]	0	0	1	1	1	1	1	0	1	1	1	8
(De Andrade, 2004) [33]	0	0	1	1	1	1	1	0	1	1	1	8
(Brennan, 2004) [27]	1	1	1	1	1	1	1	0	1	1	1	10
(Asomaning, 2008) [39]	1	1	1	1	1	1	1	0	1	1	1	10
(Olivo, 2009) [30]	1	1	1	1	1	1	1	0	1	1	1	10
(Liu, 2014) [35]	1	1	1	1	1	1	1	0	1	1	1	10
(Wang, 2015) [28]	1	1	1	1	1	1	1	0	1	1	1	10
(Bastian, 2016) [29]	0	0	1	1	1	1	1	0	1	1	1	8
(Abdelrahman, 2020) [38]	1	1	1	1	1	1	1	0	1	1	1	10
(Zhang, 2023) [55]	1	1	1	1	1	1	1	0	1	1	1	10
(Erhunmwunsee, 2022) [36]	1	1	1	1	1	1	1	0	1	1	1	10
(Choi, 2023) [37]	1	1	1	1	1	1	1	0	1	1	1	10

## Appendix E. Meta-Analysis Findings

**Table A4:** Meta-Analysis Findings.

	Reference	Sample Size	Effect Size	CI Lower	CI Upper	Ses	Weights	Stats
**Overall exposure**	**(Correa, 1983)** [23]	1338	3.11	1.02	6.45	0.47	4.52	Combined effect size 1.08 (0.96–1.22) Number of studies 8 Degrees of freedom 7 *p*-value 0.003 Cochran’s Q 21.26 I^2^ (heterogeneity) 67.08%
	**(Garfinkel, 1985)** [24]	134	1.28	1.04	3.22	0.29	12.03	
	**(Janerich, 1990)** [25]	191	1.14	1.08	2.29	0.19	27.20	
	**(Brownson, 1992)** [26]	432	0.8	0.66	1.01	0.11	84.89	
	**(Alavanja, 1995)** [40]	618	1.6	1.11	2.20	0.35	8.35	
	**(Brennan, 2004)** [27]	665	1.32	1.19	2.82	0.22	20.64	
	**(Wang, 2015)** [28]	36135	1.74	1.02	3.65	0.33	9.45	
	**(Zhang, 2023)** [55]	6508	1.045	0.84	1.29	0.19	27.42	
**Household exposure**	**(Correa, 1983)** [23]	1338	2.07	1.63	2.63	0.12	67.14	Combined effect size 1.41 (1.31–1.52) Number of studies 11 Degrees of freedom 10 *p*-value <.001 Cochran’s Q 51.57 I^2^ (heterogeneity) 80.61%
	**(Garfinkel,1985)** [24]	134	1.31	1.01	1.70	0.13	56.68	
	**(Janerich, 1990)** [25]	191	0.89	0.67	1.18	0.14	47.97	
	**(Stockwell, 1992)** [32]	210	1.60	1.25	2.05	0.13	62.79	
	**(Brownson, 1992)** [26]	432	0.70	0.52	0.95	0.15	42.31	
	**(Alavanja, 1995)** [40]	618	1.80	1.41	2.30	0.12	64.18	
	**(Cardenas, 1997)** [34]	19549	1.50	1.16	1.93	0.13	59.29	
	**(Brennan, 2004)** [27]	665	1.24	0.95	1.61	0.13	55.22	
	**(Asomaning, 2008)** [39]	138	1.29	0.99	1.67	0.13	56.21	
	**(Abdel-Rahman, 2020)** [38]	49569	1.81	1.42	2.31	0.12	64.90	
	**(Brennan, 2004)** [27]	37650	1.40	1.08	1.81	0.13	57.63	
**Work exposure**	**(Brennan, 2004)** [27]	665	1.26	1.01	1.9	0.16	38.48	Combined effect size 1.24 (1.15–1.34) Number of studies 4; Degrees of freedom 3 *p*-value 0.165; Cochran’s Q 5.09 I^2^ (heterogeneity) 41.05%
	**(Liu 2014)** [35]	650	1.22	1.17	1.37	0.04	617.05	
	**(Abdel-Rahman, 2020)** [38]	49569	2.04	1.31	3.164	0.22	19.76	
	**(Erhunmwunsee, 2022)** [36]	37650	1.29	0.74	2.26	0.28	12.32	
**Childhood exposure (both)**	**(Janerich, 1990)** [25]	191	2.07	1.4	3.06	0.20	25.13	Combined effect size 1.40 (1.15–1.70) Number of studies 4; Degrees of freedom 3 *p*-value 0.0002; Cochran’s Q 19.69 I^2^ (heterogeneity) 84.77%
	**(Brownson, 1992)** [26]	432	0.8	0.54	1.18	0.20	25.15	
	**(Olivo, 2009)** [30]	796	2.25	1.52	3.33	0.20	24.98	
	**(Liu, 2014)** [35]	49569	1.028	0.69	1.52	0.21	24.64	
**Exposure (mother)**	**(Correa, 1983)** [23]	1338	1.66	1.03	1.94	0.161	38.33	Combined effect size 1.73 (1.40–2.14) Number of studies 3; Degrees of freedom 2 *p*-value 0.24; Cochran’s Q 2.80 I^2^ (heterogeneity) 28.48%
	**(Stockwell, 1992)** [32]	210	1.6	0.99	1.85	0.159	39.31	
	**(Olivo, 2009)** [30]	796	2.92	1.12	4.11	0.331	9.09	
**Exposure (father)**	**(Correa, 1983)** [23]	1338	1.04	0.85	1.55	0.15	42.57	Combined effect size 1.22 (1.01–1.55) Number of studies 3; Degrees of freedom 2 *p*-value 0.02; Cochran’s Q 7.15 I^2^ (heterogeneity) 72.04%
	**(Stockwell, 1992)** [32]	210	1.2	0.64	1.89	0.27	13.1	
	**(Olivo, 2009)** [30]	796	2.89	1.12	4.42	0.35	8.15	
